# Inhibition of NLRP3 and Golph3 ameliorates diabetes-induced neuroinflammation *in vitro* and *in vivo*

**DOI:** 10.18632/aging.204363

**Published:** 2022-11-15

**Authors:** Yuan Li, Haifeng Zhang, Weihong Long, Menghan Gao, Weiying Guo, Lu Yu

**Affiliations:** 1 State Key Laboratory for Zoonotic Diseases, Key Laboratory for Zoonosis Research of the Ministry of Education, Institute of Zoonosis, College of Veterinary Medicine, Department of Endocrinology and Department of Interventional Therapy of First Hospital of Jilin University, Jilin University, Changchun 130000, China; 2 Innovation Pharmaceutical Research Institute of Shijiazhuang No. 4 Pharmaceutical Co., Ltd., Hebei Guangxiang Pharmaceutical Co., Ltd., Shijiazhuang 050000, China

**Keywords:** neuroinflammation, Golgi stress, NLRP3, Golph3

## Abstract

Golgi stress has been observed in various neurodegenerative diseases, such as Alzheimer’s disease and Parkinson’s disease. Whether Golgi stress participates in hyperglycemia-induced neuroinflammation, and how it is regulated remain unclear. First, we found that high glucose (HG) could induce dispersed Golgi apparatus (GA) in BV2 cells, which can be reversed by knockout of NLRP3. Next, we discovered that HG could promote the interaction of NLRP3 and VPS35 and then enhances the interaction of VPS35 and Golph3; knockout of NLRP3 suppressed the expression of VPS35 and Golph3; knockout of VPS35 reduced the expression of Golph3 but not NLRP3, indicating that HG induced the activation of NLRP3/VPS35/Golph3 pathway in BV2 cells. Further, we elucidated the signaling pathway that Golph3 mediated GA stress in HG condition. We used GST-pulldown and Co-IP experiments methods to show that HG promoted the interaction of Golph3 and Vimentin, knockout of Golph3 alleviated the expression of Vimentin. Knockout out of Golph3 and Vimentin both ameliorated HG induced dispersed Golgi apparatus. Collectively, our study demonstrated that HG regulates GA stress through NLRP3/VPS35/Golph3/Vimentin pathway. At last, we found that a combination of small molecular inhibitors targeting NLRP3 and Golph3 selected by molecular docking could alleviate HG-induced neuroinflammation *in vitro* and *in vivo.* Therefore, the molecular inhibitors targeting NLRP3 and Golph3 have great potential for use in the development of anti-diabetes neuroinflammatory therapies.

## INTRODUCTION

The Golgi apparatus (GA) is a dynamic organelle that plays important roles in protein sorting, transport and secretion under physiological conditions [1]. Under stress and pathological conditions, such as oxidative stress, inflammation and neurodegenerative diseases, the GA has to change its structure and physiological functions to counteract oxidative stress and achieve ionic homeostasis by upregulating the transcription of Golgi-related genes, a process known as “GA stress” [2, 3]. During GA stress, the integrated structure and function of the GA could be impaired, which can lead to Golgi fragmentation and cell apoptosis [4, 5]. Therefore, targeting GA stress has become a new strategy for revealing the pathogenesis of oxidative stress-associated diseases.

Diabetes neuroinflammation is the most common complication in diabetic individuals, but the pathogenic mechanism remains unclear. Studies have shown that hyperglycemia is an important inducing factor that can exacerbate oxidative stress and promote the release of cytokines [6]. Whether GA stress promotes the development of diabetic neuroinflammation is unclear. Golph3 has been reported to regulate Golgi morphology and secretory functions. Golph3 localizes to the trans-Golgi via direct interactions with PtdIns(4)P, and Golph3 also binds tightly to the unconventional myosin 18 A (MYO18A) to maintain efficient forward trafficking [7]. Golph3 has also been indicated to be an oncogene, as demonstrated by fragmented and dispersed GA that is triggered by DNA damage, which requires direct phosphorylation of the Golph3 signaling pathway [8, 9]. A recent study showed that Golph3 also mediates the GA stress response in N2A cells upon oxygen-glucose deprivation and reoxygenation injury [10], indicating that Golph3 may sense cell stress caused by disturbances in the Golgi. Whether the Golph3 pathway plays a role in the pathophysiology of diabetes neuroinflammation has not been studied.

NLRP3 has been widely studied in inflammation and neurodegenerative diseases, can be activated by various stimuli and has been implicated in innate immune diseases [11–13]. In our previous studies, we found that HG could induce NLRP3 inflammasome activation in BV2 cells and the cortex and hippocampus of streptozotocin (STZ)-induced diabetic mice [14]. We also found that overexpression of TREM2 promotes NLRP3 inflammasome activation in HG-induced BV2 cells [15].

In present study, we aim to investigate the molecular pathway of high glucose induced GA stress in diabetes-associated neuroinflammation and try to ameliorate diabetes associated neuroinflammation by targeting NLRP3 inflammasomes and GA stress through specific inhibitors that were selected by molecular docking.

## RESULTS

### NLPR3 involves HG induced GA stress

**Figure 1:**
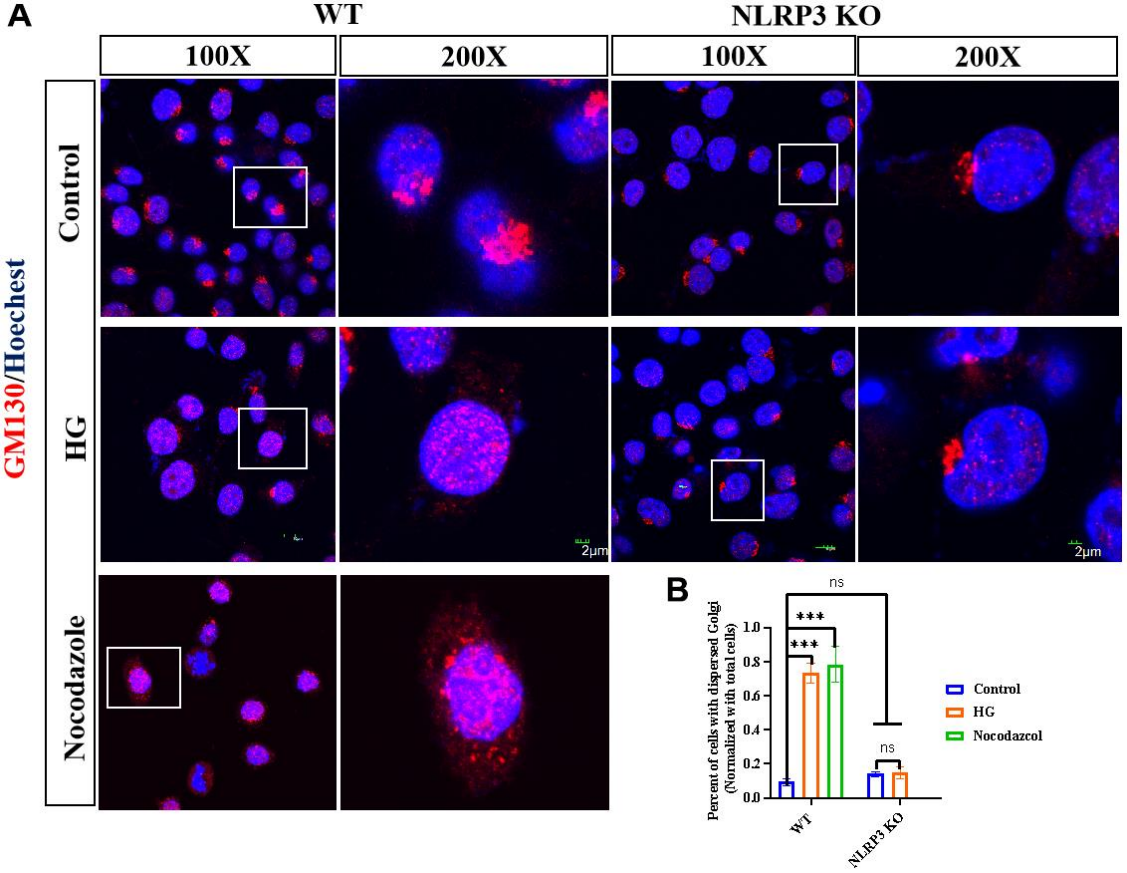
**NLPR3 involves HG induced GA stress.** (**A**) WT BV2 cells were treated with or without HG (35 mM) for 12h; WT BV2 cells were treated with nocodazole (10 μg/kg) for 5h, NLRP3 KO BV2 cells were treated with or without HG for 12 h, GM130 was detected by immunofluorescence. (**B**) The percent of cells with dispersed Golgi apparatus normalized with total cells were calculated. Bar = 2 μm. Data represent means ± SEM of 3 independent experiments. * *p* ≤ 0.05, ** *p* ≤ 0.01, and *** *p* ≤ 0.001 according to two-way ANOVA with Bonferroni’s post hoc test.

To observe whether HG could cause disturbances in the GA, we examined the expression and distribution of GM130, a Golgi marker. The Golgi stress inducer nocodazole was used as a positive control. The immunofluorescence results showed that in the control groups, the GA was compressed around the nucleus, while in the nocodazole and HG groups, the GA was dispersed around the cytoplasm, whereas NLRP3 KO ameliorated the dispersed GA in the HG group, indicating that NLRP3 KO could block HG-induced GA dispersion (Figure 1A, 1B).

### HG promotes the activation of NLRP3/VPS35/Golph3 pathway

**Figure 2:**
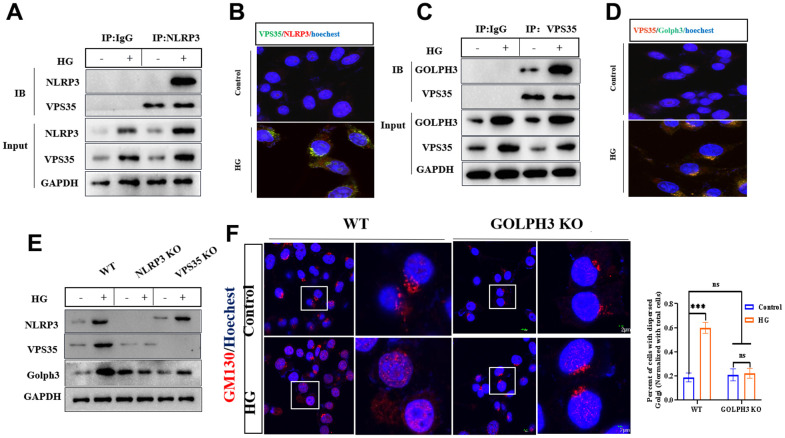
**HG promotes the activation of NLRP3/VPS35/Golph3 pathway.** (**A**) The interaction of NLRP3 and VPS35 was determined by co-immunoprecipitation experiment. Lysates from BV2 microglial cells were immunoprecipitated with a NLRP3 antibody or rabbit IgG and immunoblotted with VPS35 and NLRP3 antibody. (**B**) The colocalization of NLRP3 and VPS35 was tested by immunofluorescence method. Bar = 2 μm. (**C**) The interaction of VPS35 and Golph3 was determined by co-immunoprecipitation experiment. Lysates from BV2 microglial cells were immunoprecipitated with a VPS35 antibody or rabbit IgG and immunoblotted with VPS35 and Golph3 antibody. (**D**) The colocalization of VPS35 and Golph3 was tested by immunofluorescence method. Bar = 2 μm. (**E**) Western blot analysis of NLRP3, VPS35, and Golph3 in WT, NLRP KO and VPS35 KO BV2 cells treated with or without HG. GAPDH was used as an internal control for normalization. (**F**) Immunofluorescence detection of GM130 in WT and Golph3 KO BV2 cells, the percent of cells with dispersed Golgi apparatus normalized with total cells. Bar = 2 μm. Data represent means ± SEM of 3 independent experiments. * *p* ≤ 0.05, ** *p* ≤ 0.01, and *** *p* ≤ 0.001 according to two-way ANOVA with Bonferroni's post hoc test.

NLRP3 was reported to be recruited to the dispersed Golgi apparatus before activation [16] and Golph3 was reported to regulate Golgi stress [10] whereas VPS35 is responsible for protein transportation [17]. We found that HG promoted the interaction of NLRP3 and VPS35 by co-immunoprecipitation and immune-fluorescence methods (Figure 2A, 2B), this indicated that VPS35 was interacted with NLRP3. Next, we found that HG promoted the interaction of VPS35 and Golph3 (Figure 2C, 2D), knockout of NLRP3 suppressed the expression of VPS35 and Golph3 and knockout of VPS35 reduced the expression of Golph3 but not NLRP3, these results demonstrated that VPS35 was activated downstream of NLRP3, Golph3 was activated downstream of VPS35 under HG condition (Figure 2E). Additionally, knockout of Golph3 alleviated HG induced Golgi stress (Figure 2F). Collectively, our results suggested that HG promoted the activation of GA stress in a NLRP3/VPS35/Golph3 pathway.

### Golph3 regulates GA stress by interacting with vimentin

**Figure 3:**
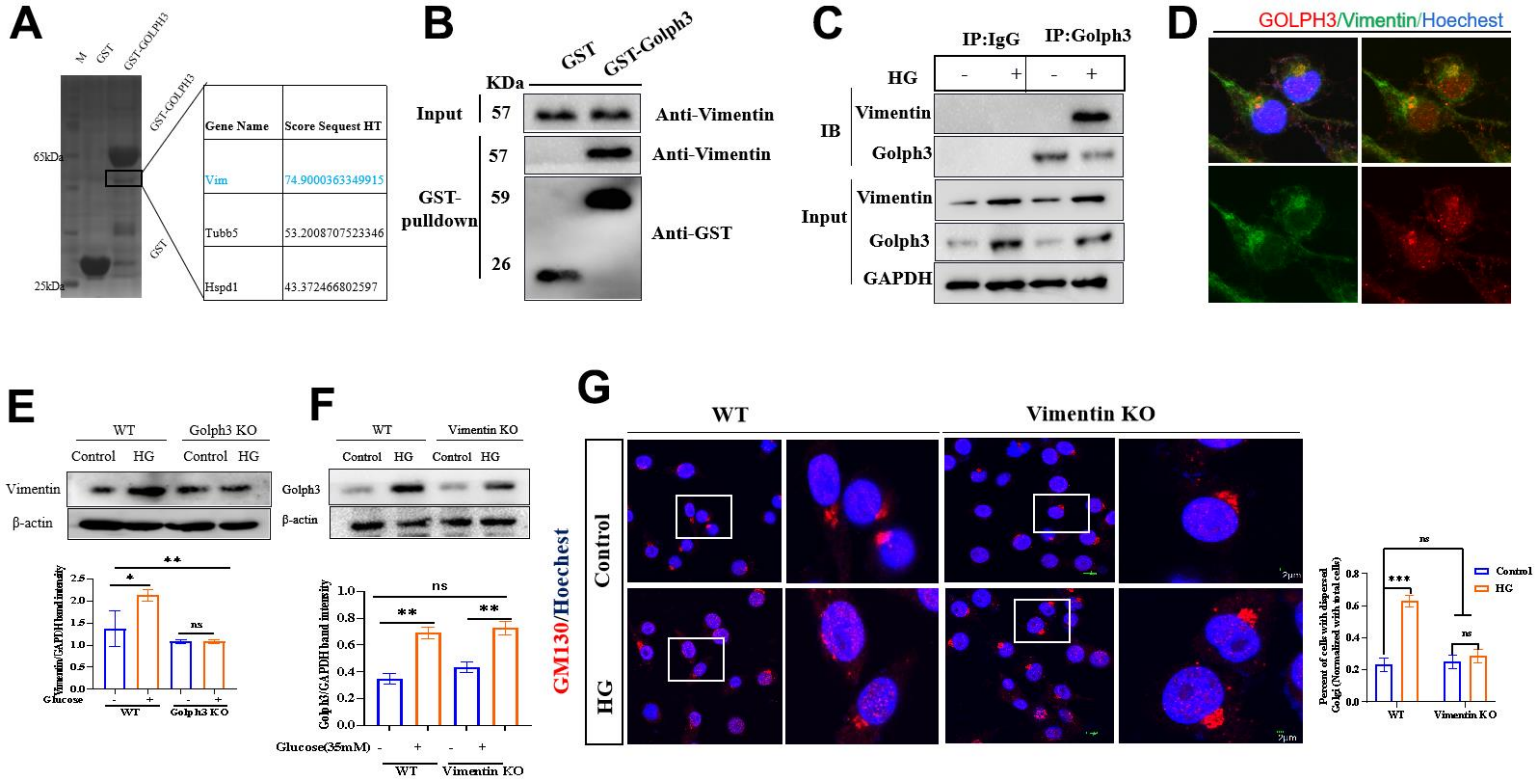
**Golph3 regulates GA stress by interacting with vimentin.** GST-Golph3 protein was expressed and purified from the *Escherichia coli* expression system, and cell lysates were precipitated with glutathione Sepharose beads and immunoblotted by SDS-PAGE, the obviously precipitated proteins in the SDS gel were tested by mass spectrometry (**A**) and immunoblotted with Vimentin antibody (**B**). (**C**) Lysates from BV2 microglial cells were immunoprecipitated with a Golph3 antibody or rabbit IgG and immunoblotted with a mouse vimentin antibody. (**D**) The colocalization of NLRP3 and VPS35 was tested by immunofluorescence method. Bar = 2 μm. (**E**) Western blot analysis of Vimentin in WT, Golph3 KO BV2 cells treated with or without HG. GAPDH was used as an internal control for normalization. (**F**) Western blot analysis of Golph3 in WT, Vimentin KO BV2 cells treated with or without HG. GAPDH was used as an internal control for normalization. (**G**) Immunofluorescence detection of GM130 in WT and Vimentin KO BV2 cells, the percent of cells with dispersed Golgi apparatus normalized with total cells. Bar = 2 μm. Data represent means ± SEM of 3 independent experiments. * *p* ≤ 0.05, ** *p* ≤ 0.01, and *** *p* ≤ 0.001 according to two-way ANOVA with Bonferroni's post hoc test.

To clarify the molecular pathway of HG induced Golph3 activation, we performed the pulldown assay with GST-tagged Golph3 mixed with BV2 cell lysate, and the isolated proteins were analyzed by mass spectrometry and immunoblot. The mass spectrometry results showed that vimentin was pulled down by GST-tagged Golph3 protein (Figure 3A). The immunoblot results also showed that vimentin interacted with Golph3 (Figure 3B). Next, we performed an immunoprecipitation assay to evaluate the cellular interaction between Golph3 and vimentin, and the results showed that vimentin could be precipitated by Golph3 (Figure 3C). The confocal microscopy results further confirmed that Golph3 colocalized with vimentin after treatment with HG (Figure 3D). Vimentin has been reported to regulate Golgi function, and increased vimentin expression could lead to dispersion of the GA [18]. Knockout of Golph3 reduced the expression of vimentin (Figure 3E), while knockout of vimentin could not influence HG induced expression of Golph3, indicating that vimentin was stimulated downstream of Golph3 (Figure 3F). In addition, knockout of vimentin also alleviated HG induced GA stress as indicated by reduced GA dispersal (Figure 3G). These results indicated that Golph3 regulates GA stress by interacting with vimentin. Collectively, we concluded that HG induced GA stress was mediated by NLRP3/VPS35/Golph3/vimentin pathway.

### Combination of NLRP3 inhibitor and Golph3 inhibitor ameliorates HG-caused microglia neuroinflammation

**Figure 4:**
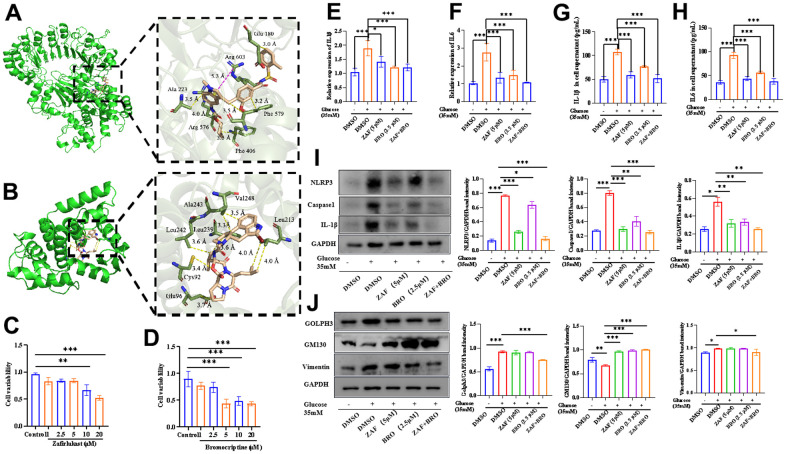
**Combination of NLRP3 inhibitor and Golph3 inhibitor ameliorates HG-caused microglia neuroinflammation.** (**A**) The 3D structure of NLRP3 with zafirlukast; (**B**) The 3D structure of Golph3 with bromocriptine, the structure of zafirlukast/NLRP3 complex and Golph3/bromocriptine for molecular dynamic simulation were obtained by a standard docking procedure for a rigid protein and a flexible zafirlukast performed with Autodock 4.0, the image was analyzed by Pymol software. (**C**) The cell viability of BV2 treated with zafirlukast detected by CCK8 kit; (**D**) The cell viability of BV2 treated with bromocriptine detected by CCK8 kit; (**E**, **F**) qPCR detection of the IL-1β and IL-6 transcription level of BV2 cells treated with ZAF, BRO, and combination of ZAF and BRO under HG condition. (**G**, **H**) ELISA detection of IL-1β and IL-6 in BV2 cells treated with ZAF, BRO, and combination of ZAF and BRO under HG condition. (**I**) Western blot analysis of NLRP3, caspase1 and IL-1β in BV2 cells treated with ZAF, BRO, and combination of ZAF and BRO under HG condition. (**J**) Western blot analysis of Golph3, GM130, Vimentin in BV2 cells treated with ZAF, BRO, and combination of ZAF and BRO under HG condition. GAPDH was used as an internal control for normalization. Data represent means ± SEM of 3 independent experiments. * *p* ≤ 0.05, ** *p* ≤ 0.01, and *** *p* ≤ 0.001 according to two-way ANOVA with Bonferroni's post hoc test.

We used virtual molecular docking to select specific inhibitors targeting NLRP3 and Golph3, hoping to develop new drug combinations to alleviate neuroinflammation caused by HG. The molecular modeling analysis performed by Autodock vina and Autodock 4.0 showed that zafirlukast is the potential inhibitors of NLRP3 and bromocriptine is the potential inhibitors of Golph3 (Table 1). The binding mode of zafirlukast and NLRP3 is shown in Figure 4A. Obviously, the hydrogen bonding and hydrophobic interactions were observed for the binding of zafirlukast to NLRP3. In detail, six residues, Ala223, Arg603, Glu180, Arg576, Phe579, and Phe406, made a strong total binding energy contribution (ΔE total ≤ -1.0 kcal/mol), suggesting that these six residues are key residues for zafirlukast binding. From the Table 1, we can see bromocriptine with the smallest binding scores resulted from both Autodock vina and Autodock 4.0. The interactions between bromocriptine and Golph3 and the residues of the binding sites are shown in Figure 4B. The seven residues Ala243, Val248, Leu242, Leu239, Leu213, Cys92, and Glu96 forming the hydrogen bonding and hydrophobic interactions with the total binding energy contribution (ΔE total ≤ -1.0 kcal/mol) made a strong binding mode of bromocriptine and Golph3.

**Table 1:** Docking scores of NLRP3 and Golph3 inhibitors.

**Targets**	**Small molecular inhibitors**	**Vina score (Kcal/mol)**	**AutoDock score (Kcal/mol)**
NLRP3	Zafurlukast	-10.5	-158
	Albamycin	-10.6	-129
	cinvanti	-11	-127
	Cycloset	-10.3	-150
	Cromoptic	-10.1	-132
	Berubigen	-9.5	-128
	Alinia	-9.4	-120
Golph3	Bromocriptine Mesylate	-10.2	-110
	50366943	-10.3	0
	Calomist	-10.3	0
	46355	-10	-109.198
	Betalin	-10.1	-12
	50162774	-10.2	0
	50423656	-10	0
	Crystodigin	-9.2	-104.73

Then, we tested the anti-neuroinflammatory effect of ZAF and BRO on HG induced BV2 cells. The cell viability tests showed that ZAF at 5 μM and BRO at 2.5 μM have no effect on BV2 cell viability (Figure 4C, 4D). ZAF, BRO alone and combination of ZAF and BRO could reduce the transcription level of proinflammatory cytokines IL-1β and IL-6 in microglial cells (Figure 4E, 4F) and the expression level of proinflammatory cytokines IL1-β and IL-6 in BV2 cells treated with HG (Figure 4G, 4H). Moreover, ZAF, BRO alone and combination of ZAF and BRO could reduce the expression level of NLRP3, caspase1, IL-1β in NLRP3 inflammasome pathway (Figure 4I) and the expression level of Golph3, GM130, and vimentin in GA stress pathway (Figure 4J).

### Combination of NLRP3 inhibitor and Golph3 inhibitor ameliorates STZ-induced diabetes neuroinflammation

**Figure 5:**
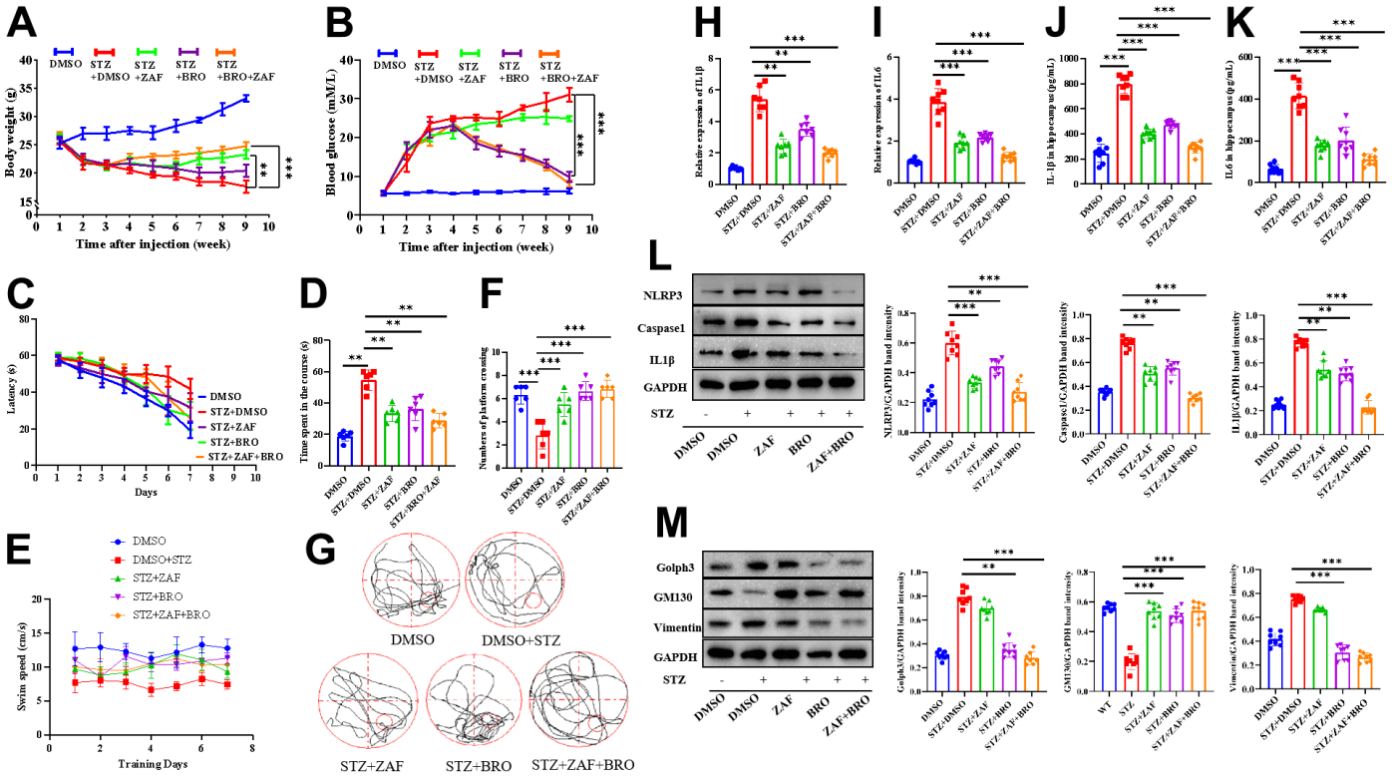
**Combination of NLRP3 inhibitor and Golph3 inhibitor ameliorates STZ-induced diabetes neuroinflammation.** Diabetes mice were constructed by injection of STZ at 70 mg/kg every other day for 3 times, the body weight and blood glucose were record every week for 4 weeks, then the mice were given ZAF, BRO, or ZAF and BRO, the mice in control group were given DMSO every day for 5 weeks, the body weight and blood glucose were record every week for 9 weeks (**A**, **B**). (**C**) Average time spent to locate submerged escape platform during the 7-d training session in the Morris water maze (n = 8/group). (**D**) Average time spent on the platform and in the four quadrant areas when the platform was absent (n = 8/group). (**E**) Average speed spent on the platform and in the four quadrant areas when the platform was absent (n=8/group). (**F**) Average numbers of mice crossing over the platform area(n=8/group). (**G**) Representative paths of mice on platform and in the four quadrant areas when the platform was absent (n = 8/group). (**H**, **I**) qPCR detection of the IL-1β and IL6 transcription level of mice hippocampus tissue treated with ZAF, BRO, and combination of ZAF and BRO. (**J**, **K**) ELISA detection of IL-1β and IL6 in mice hippocampus tissue supernatant treated with ZAF, BRO, and combination of ZAF and BRO. (**L**) Western blot analysis of NLRP3, caspase1 and IL-1β mice hippocampus tissue treated with ZAF, BRO, and combination of ZAF and BRO. (**M**) Western blot analysis of Golph3, GM130, Vimentin in mice hippocampus tissue treated with ZAF, BRO, and combination of ZAF and BRO. GAPDH was used as an internal control for normalization. Data represent means ± SEM of 3 independent experiments. * *p* ≤ 0.05, ** *p* ≤ 0.01, and *** *p* ≤ 0.001 according to two-way ANOVA with Bonferroni's post hoc test.

To test the effect of ZAF, BRO and the combination of ZAF and BRO on diabetes neuroinflammation *in vivo*, we built STZ-induced diabetes mice (Figure 5A, 5B) and evaluated the therapeutical effect of ZAF, BRO and the combination of ZAF and BRO. First, we investigated the learning behavior of mice treated with ZAF or BRO, or ZAF combined with BRO by the Morris maze test. We found that diabetes mice treated with combination of ZAF and BRO demonstrated significantly increased associative and spatial learning compared with the diabetes untreated group as indicated by the latency of mice successfully finding the targets (Figure 5C). The motor performance as indicated by time spent in the course showed that STZ induced diabetes mice spent longer time in finding the targets in the acquisition course, while, mice in ZAF, BRO, or combination of ZAF and BRO treatment group showed shorter time in finding the targets (Figure 5D). The swimming speed of mice in ZAF, BRO, or combination of ZAF and BRO were significantly faster than diabetes untreated group (Figure 5E). Moreover, ZAF, BRO and ZAF combined with BRO treatment mice showed better performance as measured by the number of crossing over the platform area (Figure 5F, 5G).

Next, we found that ZAF, BRO and the combination of ZAF and BRO reduced the transcription and expression level of proinflammatory cytokine IL-1β and IL-6 of the hippocampus of diabetes mice, respectively (Figure 5H–5K). ZAF, BRO and the combination of ZAF and BRO respectively reduced the expression level of NLRP3 pathway related protein NLRP3, caspase1 and IL-1β (Figure 5L), also suppressed the expression of Golph3 pathway related proteins Golph3, GM130 and vimentin of the hippocampus of diabetes mice, respectively (Figure 5M).

**Figure 6:**
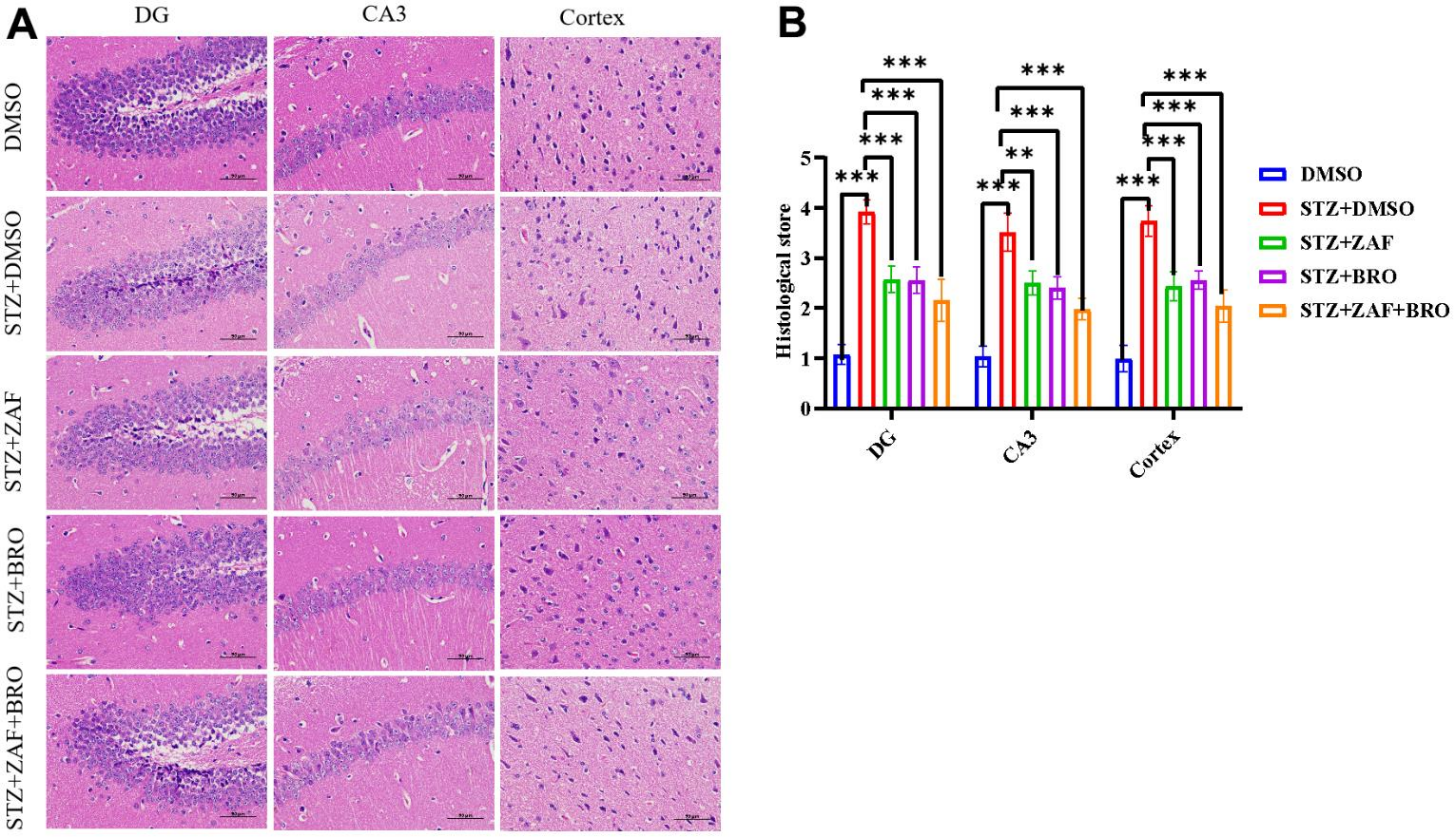
**H&E stain of brain tissues of mice treated with NLRP3 inhibitor and Golph3 inhibitor.** (**A**) H&E stain of cortex and hippocampus of mice in DMSO, STZ + DMSO, STZ + ZAF, STZ + BRO, STZ + ZAF + BRO treated groups. (**B**) Histological score of cortex and hippocampus of mice in of mice in DMSO, STZ + DMSO, STZ + ZAF, STZ + BRO, STZ + ZAF + BRO groups. All data are presented as means ± SEM (n = 8/group). Bar=50μm. * *p* < 0.05 and ** *p* < 0.01 compared with control group.

Moreover, we explored the effect of NLRP3 inhibitor combined with GOLPH3 inhibitor on the pathological injury of brain tissue in diabetic mice. We used H&E staining to detect and analyze the pathological changes in hippocampus DG region, CA3 region and cerebral cortex in control group and diabetic groups (Figure 6A, 6B). We found that zafirlukast combined with bromocriptine could alleviate the pathological changes of brain neurons such as vacuolation and swelling caused by diabetes, and the pathological score collected from three pathologists showed that the pathological score was significantly decreased in zafirlukast combined with bromocriptine treatment group in hippocampus and cortex sections, suggesting that zafirlukast combined bromocriptine played a protective role in diabetic induced neuro-damage and neuro-inflammation.

**Figure 7:**
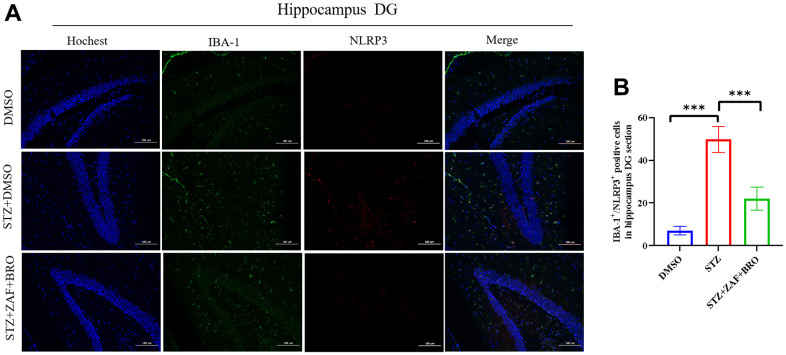
**Immunofluorescence detection of NLRP3 expression in mice hippocampus DG section treated with NLRP3 inhibitor and Golph3 inhibitor.** (**A**, **B**) Immunohistochemistry detection of NLRP3 in hippocampus DG section of mice in DMSO, STZ + DMSO, STZ + ZAF, STZ + BRO, STZ + ZAF + BRO groups. All data are presented as means ± SEM (n = 8/group). Bar=100μm. * *p* < 0.05 and ** *p* < 0.01 compared with control group.

**Figure 8:**
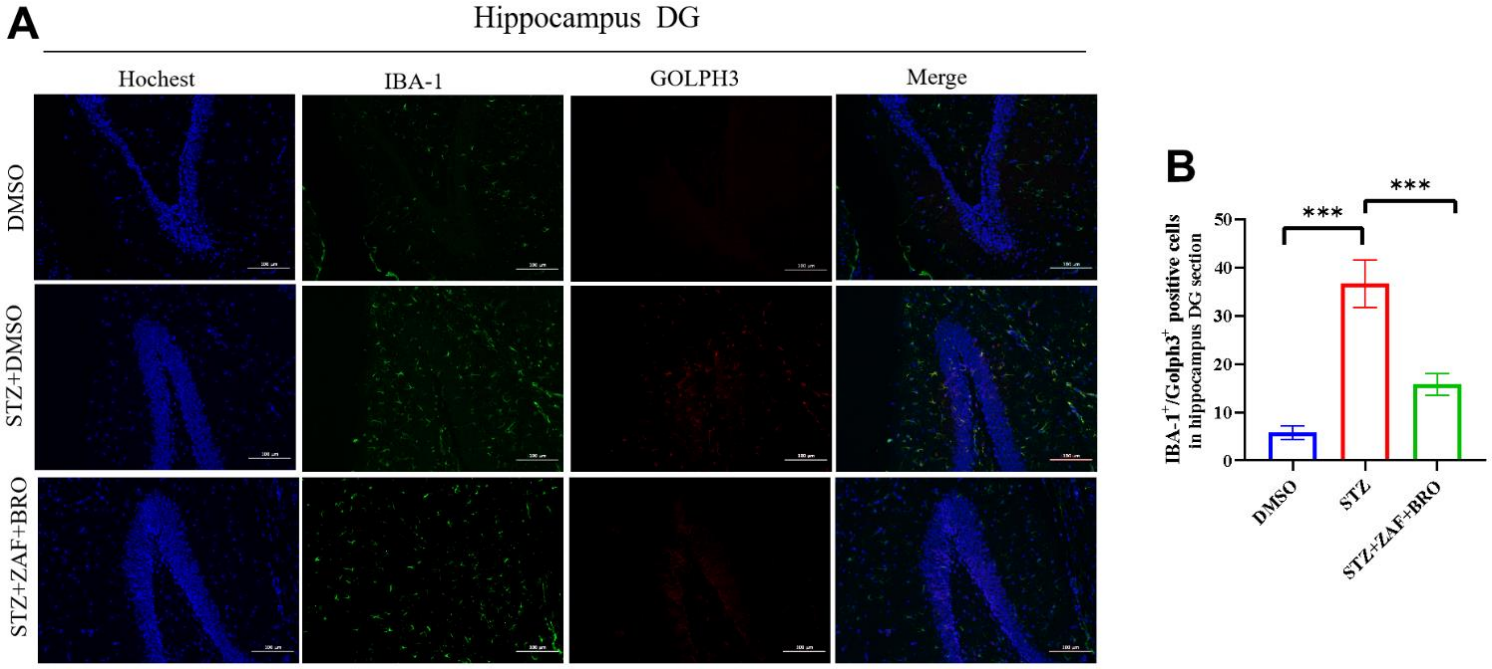
**Immunofluorescence detection of Golph3 expression in mice hippocampus DG section treated with NLRP3 inhibitor and Golph3 inhibitor.** (**A**, **B**) Immunohistochemistry detection of Golph3 in hippocampus DG section of mice in DMSO, STZ + DMSO, STZ + ZAF, STZ + BRO, STZ + ZAF + BRO groups. All data are presented as means ± SEM (n = 8/group). Bar=100μm. * *p* < 0.05 and ** *p* < 0.01 compared with control group.

At last, we determined the activation of microglia and the expression of NLRP3 and Golph3 in the treatment group by immunofluorescence method. Our results showed that the microglia were activated in STZ induced diabetes mice in hippocampus DG (Figure 7A, 7B) and CA1 (Supplementary Figures 1, 3) region, but not cortex (Supplementary Figures 2, 4), zafirlukast and bromocriptine treatment could suppress the activation of microglia in DG and CA1 regions indicated by the expression of microglia activation marker IBA1. At the same time, zafirlukast and bromocriptine treatment could suppress the expression of NLRP3 in DG (Figure 7A, 7B) and CA1 (Supplementary Figure 1) regions, and suppressed the expression of GOLPH3 in DG (Figure 8A, 8B) and CA1 (Supplementary Figure 3) regions, indicating that STZ induced diabetes might cause neuroinflammation in hippocampus regions, and zafirlukast combined with bromocriptine could ameliorate diabetes induced neuroinflammation by suppressing the expression of NLRP3 and Golph3.

In summary, these results showed that ZAF and BRO combination could ameliorate diabetes neuroinflammation *in vivo*.

## DISCUSSION

The Golgi apparatus (GA) has been studied for hundreds of years because it is the most important organelle associated with protein trafficking, processing, and sorting. The morphology and function of the GA are precisely regulated in the context of normal physiological activities, such as the cell cycle in mammalian cells, while under oxidative stress and pathological conditions, there is upregulation of GA stress-related genes [19]. GA structure and function could be impaired under stress conditions, such as DNA damage, energy and nutrient deprivation, and proapoptotic conditions, which is defined as “GA stress” [20]. GA stress has been observed in many diseases, such as pathogen infection, neurodegenerative disease, and cancer. Diabetes is considered as an immune-metabolic disease, especially diabetes-associated cognitive decline, which is caused by hyperglycemia-induced oxidative stress and inflammation in microglia that damages neurons. Whether GA stress participates in hyperglycemia-mediated induction of microglial inflammation has not been studied. It has been confirmed that oxidative stress can cause GA stress, so we explored the relationship between GA stress and hyperglycemia-induced neuroinflammation.

In our study, we first evaluated the expression of GA protein GM130 in BV2 cells upon treatment with HG and nocodazole, we found that HG could induce dispersed GA, while, NLRP3 knockout could ameliorated the dispersal of GA, then we hypothesis that NLRP3 may regulate GA stress [10]. We explored the molecular pathway through which Golph3 expression is activated by NLRP3. Co-immunoprecipitation and confocal microscopy methods were used to show that NLRP3 could activate Golph3 by directly activating VPS35. VPS35 is the main factor of the cargo-selective retromer complex that is responsible for protein cargo targeting to the Golgi apparatus or to the cell surface. Dysfunction of the VPS35 retromer complex is a risk factor for Parkinson’s disease and Alzheimer’s disease [21]. VPS35 has been reported to interact with Golph3, but its biological significance remains unclear. Golph3 has been reported to be responsible for the cell tensile force, and Golph3 binds to phosphatidylinositol 4-phosphate (PI4P) and MYO18A to create the tension required for vesicle budding and trafficking and maintenance of the Golgi ribbon [7]. Golph3 also increases cell proliferation and cell size through interactions with the retromer complex and activating mammalian target of rapamycin (mTOR) [22]. Golph3 was defined as an oncogene because it causes Golgi dispersal in response to DNA damage [23]. Moreover, Golph3 induced GA stress in an oxygen-glucose deprivation and reoxygenation model in N2A cells. Herein, we found that NLRP3 could not directly activate Golph3 but could interact with VPS35 and that depletion of VPS35 could ameliorate HG-induced GA stress. VPS35 was reported to interact with Golph3, we found that NLRP3 could regulate the activation of Golph3 by interacting with VPS35.

Next, we explored the mechanism of Golph3-mediated GA stress, pulldown, co-immunoprecipitation and confocal microscopy assays were used to show that Golph3 interacted with vimentin to exert its effect. Vimentin (VIM) is the major constituent of the intermediate filament (IF), which maintains cellular integrity and provides resistance against stress [24]. The overexpression of VIM has been reported to be correlated with epithelial cancers such as prostate cancer, CNS tumors, and breast cancer [25]. Other studies showed that the overexpression of VIM was correlated with Golgi dispersion. In our study, we found that Golph3 interacted with VIM and promoted GA stress. VIM KO ameliorated HG-induced GA stress, so we concluded that Golph3 mediates GA stress by promoting VIM expression. In summary, our results demonstrated that HG promote GA stress through NLRP3/VPS35/Golph3/Vimentin pathway.

As we known, the occurrence of diseases is a complicated process, only inhibiting the expression of one protein, often cannot achieve the effect of inhibiting the entire signal pathway. Therefore, we assumed that through inhibiting the upstream and downstream protein expression of the signaling pathway to block the signaling of HG induced Golgi stress, thus to alleviate neuroinflammation and cognitive impairment in diabetes. Therefore, we adopt the method of computer aided drug design. We simulated the 3D structure of GOLPH3 and NLRP3. The FDA approved small molecule drug library were used for the screening, in order to get their inhibitors, and to detect the inhibit effect of the combination of inhibitors on the entire signal pathway, as well as to the inflammation and the relief of diabetic cognitive impairment.

Computer aided drug design is one of the most important methods in drug development. In our study, we used Autodock vina and Autodock 4.0 to identify NLRP3 inhibitor and Golph3 inhibitor from the Drugs^@^ FDA database. From the software scoring results, we predicted that zafirlukast as the potential NLRP3 inhibitor and bromocriptine as the potential Golph3 inhibitor. Zafirlukast is a medication used in the management and treatment of chronic asthma [26]. Zafirlukast has also been reported to prevent tamoxifen-induced oxidative stress and inflammation [27] and TNF-αinduced endothelial inflammation [28]; Bromocriptine has been reported to be used for glycemic control in patients with type 2 diabetes mellitus [29]. Bromocriptine has also been studied for therapeutic applications in endocrine and neurological diseases [30]. In our study, we first investigated that zafirlukast might be an inhibitor of NLRP3 and bromocriptine might be an inhibitor of Golph3 by molecular virtual screening.

**Figure 9:**
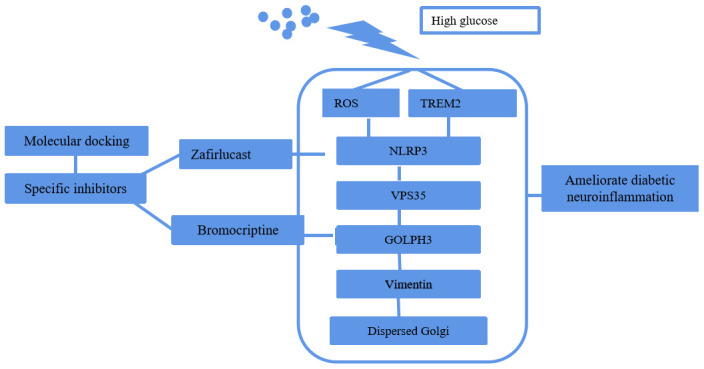
Schematic depiction of the molecular pathway of HG induced GA stress and the selection of specific inhibitors by molecular docking.

To further confirm the inhibitory effect of zafirlukast and bromocriptine, the compounds were brought and the inhibitory effect was evaluated. Our results showed that zafirlukast combined with bromocriptine could inhibit the expression of NLRP3 and Golph3 and its related proteins. Moreover, zafirlukast combined with bromocriptine could suppress the inflammatory cytokines such as IL-1β and IL-6 released by HG induced microglia. Furthermore, to confirm if zafirlukast combined with bromocriptine could suppress the neuro-inflammation and ameliorate the diabetes associated cognitive decline, we constructed STZ induced type1 diabetes mice, and evaluated the anti-diabetes effect of zafirlukast and bromocriptine. Our *in vivo* study showed that the combination of zafirlukast and bromocriptine could decrease the blood glucose level of diabetes mice and improved the cognitive impairment of diabetes mice (Figure 9).

From the FDA data, bromocriptine had been approved as a supplemental medication of adults with type II diabetes mellitus to help with glycemic control [31, 32]. But the exact mechanism of the anti-diabetes effect of bromocriptine was not entirely identified. Some of the possible mechanisms of bromocriptine including increases the production of the glucose transporter Glucose transporter (GLUT2), and increases or mimics glucagon-like peptide-1 activity [33]. Others show that the anti-diabetes activity of bromocriptine may be related to its modulation action on the neurotransmitter centrally followed by improvement in the glucose tolerance and reduction of insulin resistance in diabetic rats [34]. However, bromocriptine does not have a specific receptor to mediate a direct response on glucose metabolism and the molecular mechanism of action by which bromocriptine mediates its pharmacological effect exactly needs more investigation. In our study, by molecular docking and virtual screening, we found that bromocriptine could inhibit the expression of Golph3, which mediates the HG induced Golgi stress, thus exert its protective function against diabetes neuroinflammation.

## CONCLUSIONS

Our study elucidated that HG induced Golgi stress was mediated by NLRP3/Vps35/Golph3/Vimentin pathway, inhibition of NLRP3 and Golph3 by selected molecular inhibitors zafirlukast and bromocriptine could suppress neuro-inflammation and Golgi stress thus ameliorate diabetes cognitive decline. Therefore, zafirlukast combined with bromocriptine have great potential for use in the development of anti-diabetic neuroinflammation therapies.

## MATERIALS AND METHODS

### Cell culture and treatment

The BV2 cell line was kept in our lab. Cells were cultured in low-glucose DMEM (5.5 mmol/L) (HyClone, USA, catalog: SH30021.01) containing 10% FBS (BI, Israel, catalog: 04-001-1ACS) and 1% penicillin-streptomycin (Gibco, USA, catalog: 15140122) at 37° C in an atmosphere of 5% CO_2_. The cells (1 × 10^6^) were treated with different concentrations of D-glucose (Sigma, USA, catalog: 154-17-6) for 12h.

### Establishment of diabetes mice and drug treatment

Forty male C57BL/6 mice (6 ~ 8 weeks old) were purchased from the Experimental Animal Center of First Hospital of Jilin University. For diabetes mice model, 40 C57BL/6 mice were divided into 5 groups, 8 mice in each group. The mice from diabetic group were administered with streptozotocin (STZ, Sigma, USA) intraperitoneally at the dose of 70 mg/Kg in 0.1 M citrate buffer (CIT), and PH 4.2 ~ 4.4 every other day for 3 times. The mice from control group were injected with CIT alone and served as the vehicle controls. The blood glucose level and body weight were recorded weekly for 4 consecutive weeks. The blood glucose level was determined by a glucometer, and diabetes was confirmed by fasting blood glucose > 16.7 mmol/L. The diabetes cognitive decline was determined by the Morris maze test. Then the control group and diabetes groups were given DMSO only, the zafirlukast (Yuanye, Shanghai, China, catalog: 107753-78-6) groups were given zafirlukast at the dosage of 50 mg/kg by intraperitoneal injection, the bromocriptine groups were given bromocriptine (MedChemExpress, USA, catalog: HY-12705A) at the dosage of 12 mg/kg by intraperitoneal injection, the combination groups were given both zafirlukast and bromocriptine.

### Cas9-mediated knock out (KO) cell line construction

CRISPR/Cas9-mediated gene editing was performed using a px459 vector (Addgene, catalog: 48139) targeting murine *nlrp3*, *vps35*, *golph3*, *vimentin* in BV2 cells, the plasmid construction was described in a previous study [35]. The sgRNAs were displayed in Table 2 and were synthesized by Comate Bioscience Company, China. Plasmids were transfected with Attractene Transfection Reagent (Tiangen, China, catalog: 301004) according to the manufacturer’s instructions. Clonal lines were established by 96-well plate screening. The KO cell lines were certified by western blotting after the clones had formed. For HG treatment experiment, the KO cells were treated with HG at 35 mM for 12 h.

**Table 2:** Sequences of sgRNAs.

**Target genes**	**sgRNA sequences**
sgRNA-NRLP3	3’-AAACCTAGATACTGAGCCAGCTTGC-5’
	5’-CACCGCAAGCTGGCTCAGTATCTAG-3’
sgRNA-VPS35	3’-CACCGTCGACGTAGTCCACACGATC-5’
	5’- AAACGATCGTGTGGACTACGTCGAC-3’
sgRNA-GOLPH3	3’- CACCGCCTGGTGCAGCGGCGCACCG-5’
	5’-AAACCGGTGCGCCGCTGCACCAGGC-3’
sgRNA-Vimentin	3’-CACCGAGACTCGGTGGACTTCTCGC-5’
	5’- AAACGCGAGAAGTCCACCGAGTCTC-3’

### Immunohistochemistry and immunocytochemistry analysis

The immunohistochemistry was described as previous studies. Brain sections (30 μm) were prepared for IHC. First, the sections were made for antigen retrieval using citrate buffer, then washed with PBS for three times. After that, the sections were blocked with block buffer (5% BSA) for 1h and then cultured with primary antibody at 4° C overnight. Then the sections were washed with PBS for three times, incubated in second antibodies for 1 h at 37° C in dark, last, sections were stained with Hoechst for 10 minutes.

The immunocytochemistry study was described previously. Briefly, the cells were fixed with 4% paraformaldehyde, washed with PBS for 3 times, blocked with 5% BSA, then incubated with primary antibodies at 4° C overnight. Then cells were washed with PBS for 3 times, incubated with secondary antibody at 37° C for 1 h in dark, stained with Hoechst for 10 minutes. The cells were mounted on slides using glycerinum visualized by fluorescence microscope (Olympus U-TV0.63XC, Tokyo, Japan). The microglia were labeled with anti-IBA-1 antibody (Abcam, Beijing, China, catalog: ab178846; Diluted with 5% fetal bovine serum, 1:200), anti-NLRP3 (1:1,000), (Cell Signaling Technology, USA, catalog: 15101S); anti-Golph3 (Finetest, Wuhan, China, catalog: FNab03561), anti-GM130 (Finetest, Wuhan, China, catalog: FNab03558), anti-Vimentin (Finetest, Wuhan, China, catalog: FNab09410); Alexa Fluor dye-conjugated secondary antibodies were used for ICC experiments.

### Quantitative real-time PCR (qRT-PCR)

Total RNA was extracted from cultured cells (1 × 10^6^) using a total RNA extraction kit (QIAGEN, USA, catalog: 90001), and reverse transcription was performed using an EasyScript^®^ Reverse Transcriptase kit (TransGen Biotech, Beijing, China, catalog: AE301-02). qRT-PCR was carried out with the Applied Biosystems 7900HT fast real-time PCR system using SYBR Green PCR master mix (ROCHE, USA, catalog: 4913850001). The primers for IL-1βwere 3’-TGCCACCTTTTGACAGTGATG-5’, 5’-AAGGTCCACGGGAAAGACAC-3’; for IL6 were 3’-CCCCAATTTCCAATGCTCTCC-5’, 5’-CGCACTAGGTTTGCCGAGTA-3’. All reactions were performed in triplicate, and each experiment was repeated three times. The relative expression of each target gene was calculated using the 2^-ΔΔCt^ method.

### Western blot

The method was performed as described in a previous study [36]. The antibodies used were as follows: anti-NLRP3 (1:1,000), (Cell Signaling Technology, USA, catalog: 15101S), anti-cleaved-caspase-1 (1:1,000), (Cell Signaling Technology, USA, catalog: 89332S), anti-Cleaved-IL-1β (1:1000) (Cell Signaling Technology, USA, catalog: 63124S), and anti-GAPDH (1:2,000) (Bioworld, China, catalog: AP0066); anti-Golph3 (Finetest, Wuhan, China, catalog: FNab03561), anti-GM130 (Finetest, Wuhan, China, catalog: FNab03558), anti-Vimentin (Finetest, Wuhan, China, catalog: FNab09410); Goat anti-rabbit antibody (Bioss, Beijing, China, catalog: bs-0295G); Goat anti-mouse antibody (Bioss, Beijing, China, catalog: bs-0296G).

### ELISA

Cells (1 × 10^6^) were incubated in 6-well plates and treated with or without HG at 35 mM for 12 h. The cell supernatant was collected and the pro-inflammatory cytokine IL1β (Boster, Wuhan, China, catalog: EC0394) and IL6 (Boster, Wuhan, China, catalog: EC0394) was detected using ELISA kit according to manufactures’ instruction.

### Co-immunoprecipitation

The BV2 (1 × 10^8^) cells were collected and lysed with RIPA lysis buffer (1% NP-40, 0.25% deoxycholate) (Beyotime, China, catalog: P0013D) with 1 mM phenylmethylsulfonyl fluoride (PMSF) (Beyotime, China, catalog: ST505). 30 μg protein was added into 500 ul RIPA lysis buffer, along with 1μg portions of the corresponding antibodies, 1 μg normal rabbit IgG (Santa Cruz Biotechnology, USA, catalog: sc-2026). Then, 30 μL protein A/G (SMRRT, China, catalog: SA032005) beads were added to the protein-antibody mixture and incubated at 4° C overnight. After the incubation, samples were centrifuged at 2, 500 rpm for 4 min at 4° C and washed three times with RIPA Lysis Buffer. Next, the supernatant was removed and 30 μL of 2 × loading buffer was added boiled for 10 minutes, immunoblot (IB) analysis was performed.

### GST pull-down assay

PGEX4T-1 was purchased from PPL (Public Protein/ Plasmid Library, catalog: 27-4580-01) was used to construct p-GEX4T1-λ-Golph3 plasmid. The PCR products target *Golph3* gene were amplified from mouse cDNA library using the primers 5’-CG GAATCCATGACCTCGCTGACCCAG-3’ and 3’- CCCTCGAG TTACTTGGTAAACGCAGC-5’. The PCR products and the PGEX4T1 λ were both digested by nuclease EcoR1 (NEB, USA, catalog: R0101S) and Xho1(NEB, USA, catalog: R0146S), constructing the recombinant plasmid using T4 DNA ligase (NEB, USA, catalog: M0202S). The recombinant plasmids were transformed into *E.coli* prokaryotic expression system to obtain purified GST-tagged Golph3 protein. Purified GST or GST-Golph3 proteins were incubated with glutathione-sepharose beads (PureCube, China, catalog: 32103) at 4° C for more than 2 h. Then the beads were washed for 3 times with 1% Triton 100 (Solarbio, China, catalog: 9002-93-1). After that, the beads were added to BV2 cell lysate at 4° C for 3 h, the cell lysate were extracted from 10^8^ cells. The beads were then washed extensively and the bound proteins were eluted and separated on 10% SDS-PAGE for western blot analysis.

### Morris water maze test

The mice were individually placed into a new cage 2 days before the test. The water maze consisted of a circular tank (diameter = 1 m; height = 30 cm) that was filled with tepid water (23 ± 1° C) that was made opaque by the addition of powdered milk. The circular tank was divided into four equal sectors (Target, Opposite, Sector 1, and Sector 2), each with a spatial cue on the tank wall. A white escape platform (diameter = 10 cm, height = 10 cm) was located 1 cm below the water. The water temperature was 24 ± 1° C. The testing lasted for 7 days. First, mice were trained for 6 consecutive days and underwent four training trials per day. In each training trial, a mouse was placed in one of the sectors and allowed to search for the hidden platform for 1 min. If the mouse did not find the platform within 1 min, the experimenter led the animal to it. After the platform was located, the mouse was left on it for 15 s to memorize the spatial cues. After that, the mouse was placed in a cage for 15 s for resting before the next trial. Throughout the experiment, the platform remained at its original position. To assess the learning ability, latency to find the platform in each trial and the total number of successful attempts were registered. Finally, on day 7, a probe trial was administered: the platform was removed, the mouse was placed in the Opposite sector, and the time spent in each sector within 1 min was measured. The Morris maze equipment was kept in college of food science and engineering, Jilin University. The test data were recorded and processed using the ANY-Maze™ (Stoelting Co, Wood Dale, IL, USA).

### Virtual screening analysis and molecular docking studies

The compounds from Drugs^@^ FDA database (https://www.bindingdb.org/bind/ByFDAdrugs.jsp) were used for virtual screening. The structure of the murine NLRP3 is generated from SWISS-MODEL website (https://swissmodel.expasy.org/interactive) using the *nlrp3* sequence (NM_145827.4). The structure of the murine Golph3 is also generated from SWISS-MODEL using the *golph3* sequence (NM_025673.2). AutoDock Vina and Autodock 4.0 software was utilized in all the docking experiments, with the optimized model as the docking target. The screening method is restricted to molecular docking, and molecular dynamics simulation has not been carried out. The results were visualized using PyMol [37].

### Hematoxylin and eosin (H&E)

Mice were deeply anesthetized by Chloral Hydrate (Yuanye, Shanghai, China) and transcardially perfused with 4% of parafomaldehyde (Beyotime, China). The cortex and hippocampus of mice were carefully removed and fixed in 4% of paraformaldehyde overnight, and were decalcified in 10% of EDTA, and embedded in paraffin. Sections (4 μm) were stained with hematoxylin-eosin. The stained sections were graded by three pathologists in a blinded fashion for the degree of neuron injury and inflammation using a four-point scale from 0 to 5, and 0 represents no damage, 1-2 represents mild damage, 3-4 represents moderate damage and 5 represents severe damage, respectively. Similar results were obtained from three independent experiments.

### Statistical analysis

Data were analyzed by GraphPad Prism 8.0. Statistical significance was evaluated using independent sample one-way ANOVA or two-way ANOVA combined with post hoc tests for multiple comparisons. *p* < 0.05 was considered statistically significant.

## Supplementary Material

Supplementary Figures
