# Supplementary material for: Inhibition of NLRP3 and Golph3 ameliorates diabetes-induced neuroinflammation in vitro and in vivo
Source: Aging (Albany NY). 2022 Nov 15;14(21):8745–62. doi: 10.18632/aging.204363 (PMC9699760; doi:10.18632/aging.204363)
Supplement: Supplementary Figures [file aging-14-204363-s001.pdf]

SUPPLEMENTARY FIGURES

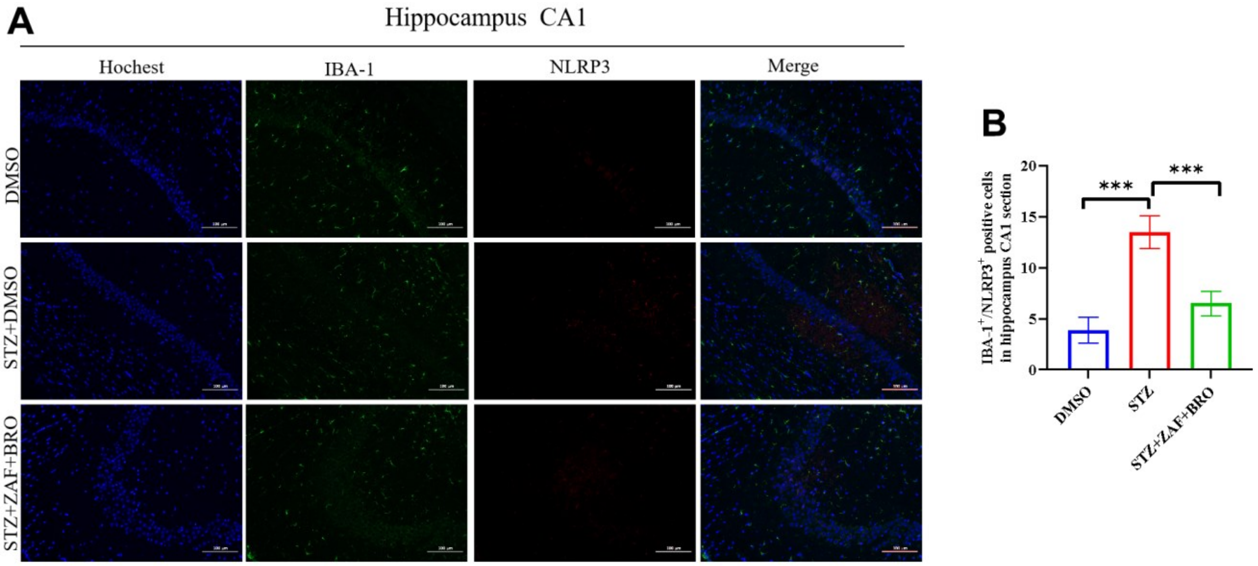

Supplementary Figure 1. Immunofluorescence detection of NLRP3 expression in mice hippocampus CA1 section treated with NLRP3 inhibitor and Golph3 inhibitor. (**A**, **B**) Immunohistochemistry detection of NLRP3 in hippocampus CA1 section of mice in DMSO, STZ + DMSO, STZ + ZAF, STZ + BRO, STZ + ZAF + BRO groups. All data are presented as means ± SEM (n = 8/group). Bar=100 μm. * $p < 0.05$ and ** $p < 0.01$ compared with control group.

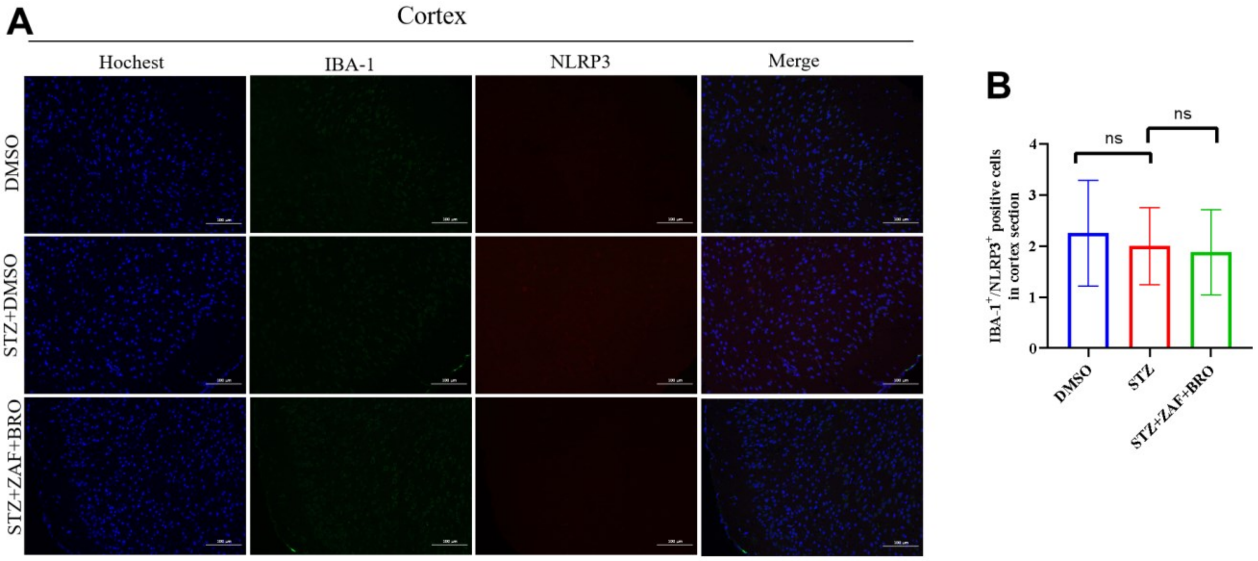

Supplementary Figure 2. Immunofluorescence detection of NLRP3 expression in mice cortex treated with NLRP3 inhibitor and Golph3 inhibitor. (**A**, **B**) Immunohistochemistry detection of NLRP3 in cortex of mice in DMSO, STZ + DMSO, STZ + ZAF, STZ + BRO, STZ + ZAF + BRO groups. All data are presented as means ± SEM (n = 8/group). Bar=100 μm. * $p < 0.05$ and ** $p < 0.01$ compared with control group.

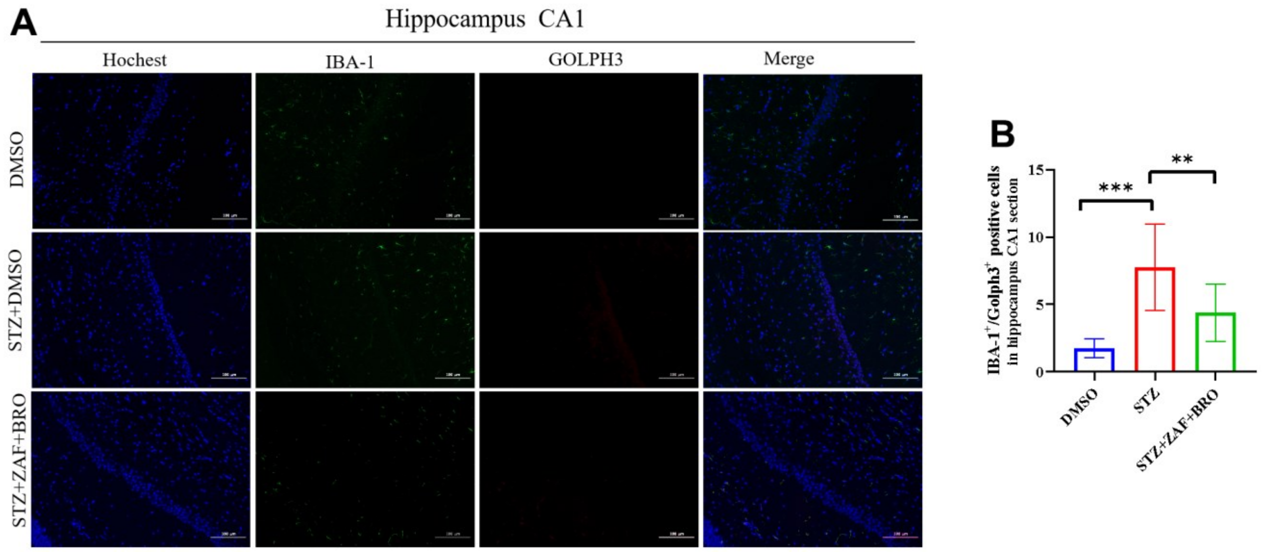

Supplementary Figure 3. Immunofluorescence detection of Golph3 expression in mice hippocampus CA1 section treated with NLRP3 inhibitor and Golph3 inhibitor. (**A**, **B**) Immunohistochemistry detection of NLRP3 in hippocampus CA1 section of mice in DMSO, STZ + DMSO, STZ + ZAF, STZ + BRO, STZ + ZAF + BRO groups. All data are presented as means ± SEM (n = 8/group). Bar=100 µm. * $p < 0.05$ and ** $p < 0.01$ compared with control group.

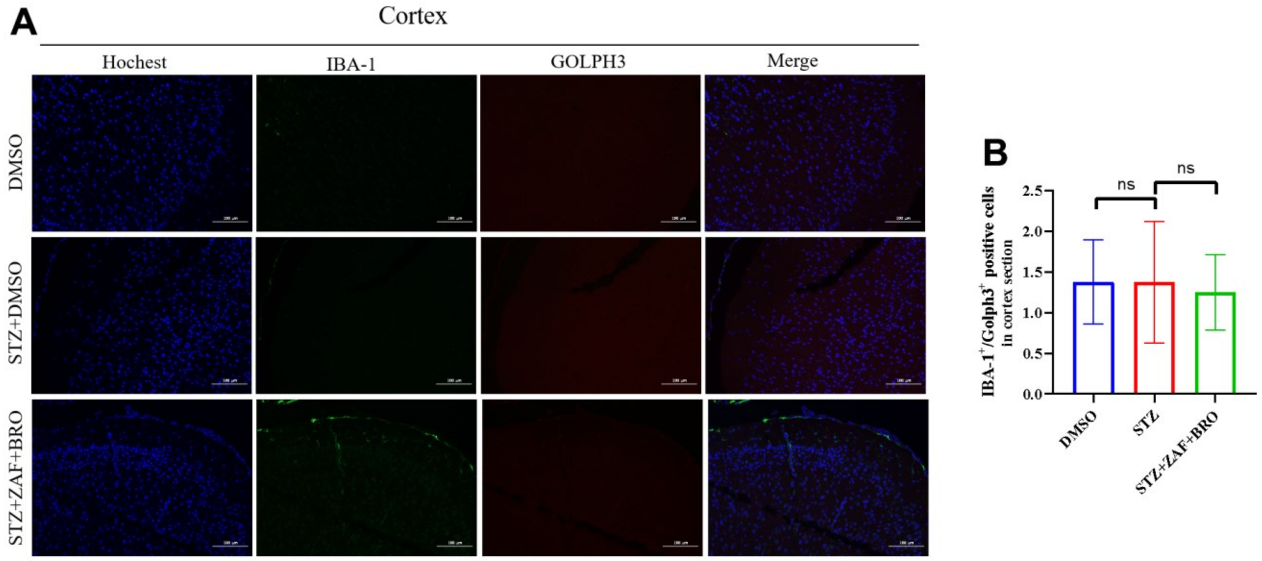

Supplementary Figure 4. Immunofluorescence detection of Golph3 expression in mice cortex treated with NLRP3 inhibitor and Golph3 inhibitor. (**A**, **B**) Immunohistochemistry detection of NLRP3 in cortex of mice in DMSO, STZ + DMSO, STZ + ZAF, STZ + BRO, STZ + ZAF + BRO groups. All data are presented as means ± SEM (n = 8/group). Bar=100 µm. * $p < 0.05$ and ** $p < 0.01$ compared with control group.
